# Increasing Permittivity and Mechanical Harvesting Response of PVDF-Based Flexible Composites by Using Ag Nanoparticles onto BaTiO_3_ Nanofillers

**DOI:** 10.3390/nano12060934

**Published:** 2022-03-12

**Authors:** Nadejda Horchidan, Cristina Elena Ciomaga, Lavinia Petronela Curecheriu, George Stoian, Mihaela Botea, Mihaela Florea, Valentin Adrian Maraloiu, Lucian Pintilie, Florin Mihai Tufescu, Vasile Tiron, Aurelian Rotaru, Liliana Mitoseriu

**Affiliations:** 1 Dielectrics, Ferroelectrics & Multiferroics Group, Faculty of Physics, Al. I. Cuza University of Iasi, Bv. Carol I, no. 11, 700506 Iasi, Romania; nadejda.horchidan@uaic.ro (N.H.); lavinia.curecheriu@uaic.ro (L.P.C.); 2 Department of Exact & Natural Sciences, Institute of Interdisciplinary Research, Al. I. Cuza University of Iasi, Bv. Carol I, no. 11, 700506 Iasi, Romania; 3 National Institute of Research and Development for Technical Physics, 700050 Iasi, Romania; gstoian@phys-iasi.ro; 4 National Institute of Materials Physics, Atomistilor 405A, 077125 Magurele, Romania; mihaela.botea@infim.ro (M.B.); mihaela.florea@infim.ro (M.F.); maraloiu@infim.ro (V.A.M.); pintilie@infim.ro (L.P.); 5 GRADIENT Srl., Str. Codrescu, no. 17, 700495 Iasi, Romania; florintufescu@gradient.ro; 6 Research Center on Advanced Materials and Technologies, Department of Exact & Natural Sciences, Institute of Interdisciplinary Research, Al. I. Cuza University of Iasi, Bv. Carol I, no.11, 700506 Iasi, Romania; vasile.tiron@uaic.ro; 7 Faculty of Electrical Engineering and Computer Science & MANSiD Research Center, Stefan Cel Mare University, 720229 Suceava, Romania; aurelian.rotaru@usm.ro

**Keywords:** Ag nanoparticles, BaTiO_3_, hybrid filler, PVDF matrix, flexible composite, permittivity, mechanical energy harvesting

## Abstract

The role of Ag addition on the structural, dielectric, and mechanical harvesting response of 20%(*x*Ag − (1 − *x*)BaTiO_3_) − 80%PVDF (*x* = 0, 2, 5, 7 and 27 vol.%) flexible composites is investigated. The inorganic fillers were realized by precipitating fine (~3 nm) silver nanoparticles onto BaTiO_3_ nanoparticles (~60 nm average size). The hybrid admixtures with a total filling factor of 20 vol.% were embedded into the PVDF matrix. The presence of filler enhances the amount of β-PVDF polar phase and the BaTiO_3_ filler induces an increase of the permittivity from 11 to 18 (1 kHz) in the flexible composites. The addition of increasing amounts of Ag is further beneficial for permittivity increase; with the maximum amount (*x* = 27 vol.%), permittivity is three times larger than in pure PVDF ($\varepsilon_{r}$ ~ 33 at 1 kHz) with a similar level of tangent losses. This result is due to the local field enhancement in the regions close to the filler-PVDF interfaces which are additionally intensified by the presence of silver nanoparticles. The metallic addition is also beneficial for the mechanical harvesting ability of such composites: the amplitude of the maximum piezoelectric-triboelectric combined output collected in open circuit conditions increases from 0.2 V/cm^2^ (PVDF) to 30 V/cm^2^ for *x* = 27 vol.% Ag in a capacitive configuration. The role of ferroelectric and metallic nanoparticles on the increasing mechanical-electric conversion response is also been explained.

## 1. Introduction

New dielectric and piezo-/pyro-/ferroelectric polymer-based materials are of particularly high interest in recent years for flexible electronics applications such as portable or wearable sensors and transducers, energy harvesting elements, implantable devices for real-time health parameters monitoring, flexible building blocks for specific industrial applications, and soft robotics, etc. [1,2,3]. To provide such functionalities, properties such as high permittivity, low losses, good electromechanical coupling, high pyro- and piezoelectric coefficients, high tunability, and ferroelectric polarization switching are required. These characteristics are typical mostly of oxide-based crystalline materials from the families of ferroelectric perovskites, such as BaTiO_3_ (BT), Pb(Zr,Ti)O_3_, or other lead-based or lead-free relaxor ferroelectrics, having high permittivity ($\varepsilon_{r}\;\sim \;10^{3}–10^{4}$), but they require a high thermal budget for processability and from a mechanical point of view they are rigid and fragile. On the other hand, ferroelectric polymers and in particular, Polyvinylidene difluoride (PVDF) with the formula –(C_2_H_2_F_2_)_n_– (which is mostly used because it is also ferroelectric and piezoelectric in specific molecular arrangements), and its co-polymers are highly flexible, i.e., they present good bending and tensile properties, they have a low mechanical impedance, low dielectric loss, and also a lower permittivity with respect to one corresponding to oxides ($\varepsilon_{r}\leq 10$) [4,5]. Their combination in hetero-structural composites with polymer matrix and inorganic fillers as active phase is natural, in order to combine the advantages of both types of materials and provide solutions for flexible electrical and electromechanical devices [6]. In such composites, multiple material combinations and micro/nanostructures and fillers with different shapes and sizes or improvements in processing were reported to enhance the permittivity and piezoelectric response and to improve the electrostatic energy storage capacity and mechanical or pyroelectric harvesting character [7,8,9,10,11]. Since the effective permittivity, piezoelectric, and ferroelectric properties were still low and the further increase of inorganic filler amount would induce poor mechanical performance (reduced flexibility and brittle character), the use of hybrid oxide-metallic nanoparticle admixtures emerged as a possible solution for a further improvement of their functional characteristics [12,13,14,15]. Permittivity as high as ~50–150 was reported for over-percolated compositions [16,17], but such values are produced as a result of the slow Maxwell Wagner uncompensated interface charges contribution and they are typical for low frequencies only (below 100 Hz). 

Nowadays, portable and wearable devices also demand efficient self-powdered electricity sources. Energy harvesting from ever-present abundant mechanical ambient vibrations (e.g., vehicle motion, water flow, wind, human body movements, etc.) generated significant interest in recent years in searching for viable energy solutions for small power applications [18,19,20] and offering a way to replace batteries, which have a negative environmental impact and require replacing/recharging procedures due to their limited lifetime. Nanoscale harvesters (nanogenerators) converting mechanical energy into electric signals are mostly based on two effects: (i) piezoelectric conversion, i.e., generation of electricity in certain materials as a result of mechanical stress due to mutual coupling between mechanical and electrical energies; such piezoelectric nanogenerators denominated as PENG were first reported by Wang et al. [21] who reported an output voltage of ~8 mV and a specific surface power of 10 pW/mm^2^; and (ii) triboelectric conversion, based on a coupling effect of contact electrification and electrostatic induction. The first triboelectric nanogenerator (denoted as TENG) was built using a combination of polymers with different electronic affinity which, when brought in contact, produced positive and negative frictional charges with an output voltage of 3.3 V and a power volume density of 10.4 mW/cm^3^ [22,23]. The nanogenerators attracted widespread attention for their ability to convert various types of low-frequency mechanical vibrations into sustainable electricity able to power small electronic devices or other off-grid electrical equipment. After the first reports, the output power of both types of devices increased by orders of magnitude due to the integration of various materials (mostly polymers but also inorganic-organic combinations of materials), device architectures, and working modes. As a general trend, the piezoelectric voltage outputs of PENGs always remained smaller in comparison with the ones of TENGs [20]. The output performances of PENGs at low mechanical impact were improved by utilizing smart composite materials formed by the combination of inorganic ferro-/piezoelectric material in polymer matrices, particularly in electroactive PVDF [24,25,26]. An enhancement of the piezoelectric harvesting output (both as open-circuit voltage and short-circuit current) was found in a direct relationship with the permittivity and polarization increase in PVDF-based composites with perovskite iodide fillers [26]. The further increase of piezoelectric output in PENGs would require electrical poling. However, such composites were reported to show several drawbacks, such as a high electrical breakdown failure rate, an increase of the leakage current, and large energy consumption [27]. As an alternative, the supplementary addition of small amounts of conductive nanofillers into the polymer matrices, which results in a polarization [28] and permittivity [29] enhancement, was recently proposed and this approach was demonstrated to be beneficial for a substantial increase of piezoelectric harvesting ability while replacing a poling step. The increase of ferroelectric polarization and piezoelectric output was considered to be created by the field concentration around the conductive nanoparticles resulting in an enhancement of the overall polar character of the compound [28,30]. Combined with ferro/piezoelectric fillers, the presence of high conductivity nanoparticles (in particular noble metals) into the flexible insulating polymer was expected to improve the carrier transport mechanism and to enhance the partial voltage and polarization degree of piezoelectric grains during the polarization process, as it was shown in [30,31]. In [31], it was observed that the use of metal-piezoelectric core-shell nanofillers generates a further enhancement of the effective field in the polymer by reducing the surface energy of piezoelectric fillers, also diminishing the leakage currents. By using 3D structures of Ag-BCTZ in a silicon rubber matrix, an output voltage of 38.6 V was produced [31]. From this point of view, it seems there is still a need to search for new combinations of hybrid fillers (metal—ferro/piezoelectric) in polymer matrices in order to increase the piezoelectric response of flexible composites. 

Some approaches were proposed to effectively increase the surface charge density to optimize the TENG device’s outputs,. Similar to PENG responses, the TENG outputs were enhanced by using conductive nanofillers (e.g., metallic or carbonaceous species) in the polymer matrix [32,33] or by the surface engineering of inorganic high-permittivity filler combined with the composite’s permittivity increase, as recently proposed in [34] where a power output surface density of 1.21 W/m^2^ was generated. It was further shown that piezo- and triboelectric generation can be exploited synergically in the same structure instead of pairing di-similar polymer-based materials, as classically used for TENGs. In this way, the functional material responds simultaneously to the action of mechanical energy sources (vibrations, compression, deflection, etc.). The two effects involve non-overlapping mechanisms in the electricity generation process: TENG is related to the functional surfaces/interfaces acting in the static electrification processes, while piezoelectricity is a bulk effect related to the crystalline volume polarisation. The combination of the two types of mechanical-to-electrical energy conversion in a single integrated system in a hybrid piezo-triboelectric device (denoted as PTENG) was proposed as a valuable method to improve the mechanical-electrical conversion efficiency and was also realized with inorganic-polymer composites [35,36]. Despite a very dynamic research field concerning these types of energy conversion in flexible polymer-based composites with various device designs, geometries, and working principles, there is still room for material design, in particular, in searching for different combinations of multicomponent hybrid systems [37]. As pointed out in recent reviews, the material design of such PTENGs has been relatively less studied to understand the relationship between the components, compositions, microstructures, and the mechanical energy conversion mechanisms and their efficiency [20,38,39].

Motivated by the above-mentioned literature results and observations, flexible composites based on electroactive PVDF polymer matrix functionalized with ferro/piezoelectric BaTiO_3_ and with Ag-BaTiO_3_ nanofillers have been produced by a facile wet synthesis method and their use as PTENGs in simple capacitive configurations were explored. Small variable amounts of Ag nanoparticles (below 27 vol.%) onto BaTiO_3_ (BT) grains were embedded into a PVDF polymer matrix with compositions below the percolation limit (i.e., the total amount of Ag-BT filler was kept constant, of 20 vol.%). The role of the Ag addition on the dielectric and mechanical harvesting response of flexible composites with formula 20%(*x*Ag − (1 − *x*)BT) − 80%PVDF, where *x* = 0, 2%, 5%, 7%, and 27% (vol.) were compared with ones of the pure PVDF polymer.

## 2. Materials and Methods

**Figure 1:**
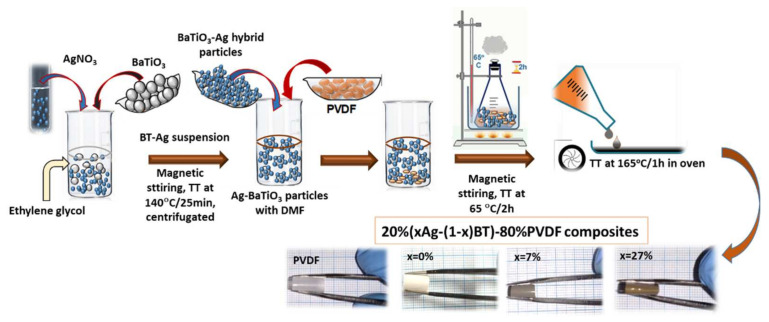
Preparation workflow for the 20%(*x*Ag − (1 − *x*)BT) − 80%PVDF flexible composites.

In the first step, *x*Ag − (1 − *x*)BT hybrid particles with variable concentrations of Ag of: *x* = 0, 2%, 5%, 7% and 27% (vol.) were prepared (Figure 1) following a similar procedure as reported in [16]. Hydrothermally synthesized BaTiO_3_ nanopowders (Sigma Aldrich, Darmstadt, Germany, purity: ≥99%) with an average particle size of ~60 nm and pseudo-cubic $Pm\bar{3}m$ symmetry were well-dispersed by magnetic stirring into a silver nitrate AgNO_3_ (Sigma Aldrich, purity 99.9999%) solution with variable desired concentrations in Ethylene glycol (Acros organics, purity 99.97%), whose role was both as a solvent for the silver precursor and as reducing agent. During the mixing at room temperature for 2 h, the silver nanocrystals started to nucleate and grow, as indicated by the change in the solution’s color.

The solution was then heated to 140 °C and maintained for 25 min to allow the complete reaction and the formation of Ag nanocrystals onto the surfaces of BaTiO_3_ templates. The mixture was centrifugated and washed three times with ethanol and then dried at 50 °C for 8 h. The stoichiometry in the final product (*x* amount) was confirmed for each composition by quantitative X-ray diffraction (XRD) analysis of the composite powders, which indicated that no synthesis product was lost by washing, and confirmed the formation of *x*Ag − (1 − *x*)BT hybrid composite powders with nominal compositions. Further, the *x*Ag − (1 − *x*)BT hybrid particles with a total filler concentration of 20 vol.% as determined by using the theoretical densities of silver of 10.49 g/cm^3^, of BT of 6.08 g/cm^3^, and PVDF of 1.11 g/cm^3^, were embedded into the PVDF polymer matrix using the following method: PVDF pellets (Sigma Aldrich, Mw = 175,000) were dissolved in N, N-Dimethylformamide (DMF) of 99.5% (Merk) under magnetic stirring at 65 °C with a mass ratio 20/80. The Ag-BT hybrid particles with desired compositions were introduced in this solution and mixed by magnetic stirring for 2 h at 65 °C to allow the perfect solubilization of PVDF. The mixture product was then cast in Petri dishes and dried for one hour at 165 °C in an oven. Flexible composite films with formula 20%(*x*Ag − (1 − *x*)BT) − 80%PVDF (volume ratios), where *x* = 0, 2, 5, 7 and 27 vol.% and thickness of about 1.5–2 μm were obtained (Figure 1).

The morphology of the template BT nanopowders and the Ag-BT hybrid inorganic composites was revealed using an analytical transmission electron microscope JEOL JEM ARM200F (JEOL Ltd., Tokyo, Japan) operating at 200 kV and equipped with a JEOL JED-2300T unit for Energy-Dispersive X-ray spectroscopy (EDX) analysis. The powders were gently crushed in an agate mortar and dispersed in ethanol. A droplet of this suspension was then deposited onto a 200-mesh carbon lacey TEM Cu grid and allowed to dry at room temperature. EDS spectra were obtained using the X-ray signal collected by the detector originating from the area of interest illuminated with a parallel electron beam. For the elemental maps, regions of interest were scanned with a convergent beam with a diameter of approximately 0.3 nm. The X-ray signal from each point of the scan was collected by the detector. The maps of the selected chemical elements, using the corresponding *k* lines, were obtained. The surface topography and local piezoelectric character were explored with a multimode AFM system (Solver Pro from NT-MDT) equipped with a PFM measurement module using commercial probes with a Pt coating (CSG30/Pt from TipsNano), the curvature radius of 35 nm, free resonance frequency *f* = 33.5 kHz, and spring constant *k* = 0.6 N/m. The Nova Software from NT-MDT was used for microscope control, data acquisition, and analysis. 

The phase structure of the starting nanopowders and the thick film nanocomposites was examined by X-ray diffraction (XRD) with a Shimadzu LabX 6000 diffractometer using Ni-filtered CuKα radiation (λ = 1.54 Å) with scan step increments of 0.02° and a counting time of 1 s/step, for 2θ ranging between (10°–80°). The fast-Fourier transformed infrared spectroscopy (FTIR) analysis of film composites was performed with a spectrometer Nicolet Impact 4000 with Monolithic Diamond as internal reflection element. The FTIR spectrum was recorded in transmittance mode in the range of 4000–400 cm^−1^ (resolution of 4 cm^−1^) with an incident angle of 45°. Room temperature Raman analysis of the composite films was performed with a LabRAM HR Evolution spectrometer, from Horiba Jobin Ivon, with the laser radiation at a wavelength of 633 nm with ~25% power during testing. The spectra were recorded in the extended scan mode in the range from 150 cm^−1^ to 1500 cm^−1^ wavenumbers. All the data were collected using five accumulations with a 30 s exposure time to ensure proper resolution of the Raman spectra. 

The low field dielectric characterization was performed in a parallel-plate capacitor configuration of the flexible composite thick films using Au sputtered electrodes (20 mm × 20 mm) applied onto their surfaces. The impedance spectroscopy characterization was carried out with an Agilent E4980A Precision LCR Meter (Agilent, Santa Clara, CA, USA) fed with a sine wave of 1.5 V amplitude in the frequency range of (100 Hz–1 MHz) at room temperature. The polarization-field responses under high voltage have been collected on the composite films immersed in silicone oil, at room temperature, with a Radiant Precision Multiferroic II Ferroelectric Test System (Radiant Technologies Inc.) using a modified Sawyer-Tower circuit using a sinusoidal waveform with a maximum amplitude of *E* = 125 kV/cm as input produced by a function generator (DS345, Stanford Res. Systems, Sunnyvale, CA, USA) coupled with a High-Voltage Amplifier (Trek 609 × 10^6^, Trek Inc., Medina, NY, USA). The *P(E)* loops were recorded in a dynamic regime, at 1 Hz, on unpoled samples. 

The mechanical-electrical conversion harvesting performance of the hybrid composite films was determined by using an in-house setup (depicted in Figure S1, Supplementary material) consisting of a programmable linear stepping dc motor used for providing periodic compressive stress on the composite samples. A periodic impact force, *F* = 0.36 N with a low frequency of 1.71 Hz, was uniformly applied to the sample surface which was placed between two Cu-electrodes on plain support. The output harvested voltage was collected under open-circuit conditions by an oscilloscope (Tektronix, TDS 2012C, Beaverton, OR, USA) with an internal impedance of 1 MΩ.

## 3. Results and Discussion

### 3.1. Microstructural Characterization

**Figure 2:**
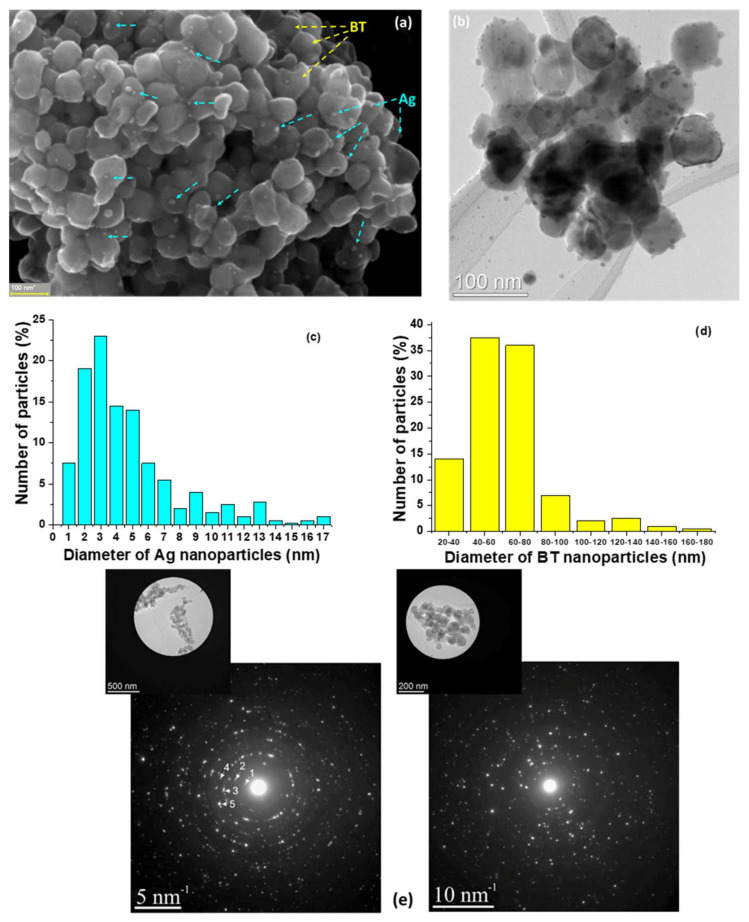
Microstructural characterization of 2%Ag-98%BT hybrid powders: (**a**) SEM micrograph for aggregated hybrid nanoparticles, (**b**) TEM image of the dispersed nanoparticles, (**c**) Particle size distribution for Ag (**c**) and BaTiO_3_ components (**d**) of the hybrid Ag-BT mixtures, and (**e**) diffraction diagrams realized on Ag-BT aggregated nanoparticles and their corresponding SAED patterns.

The microstructural details of the 2%Ag–98%BT (vol.) hybrid powders used as fillers into the PVDF matrix are presented in Figure 2. Figure 2 shows an SEM micrograph of an agglomerated region of such Ag-BT powder in which the two components can be observed: ultrafine rounded Ag nanoparticles, with sizes from a few units to a maximum of ~17 nm, very well distributed onto the surfaces of BaTiO_3_ larger grains having diameters in the range of 10–200 nm. No agglomeration of silver nanoparticles is noticed, even in the regions of larger aggregates, which means that the synthesis method allowed the formation of well-dispersed Ag nanoparticles onto the BaTiO_3_ template surfaces. This is confirmed by the Transmission Electron Microscopy (TEM) images of the hybrid Ag-BT powders (Figure 2b) showing BaTiO_3_ larger polyhedral grains with well-defined, faceted clean surfaces, uniform shape, and size. 

The chemical homogeneity of the starting BaTiO_3_ powders was demonstrated in [39] by EDX mapping and by electron energy loss spectroscopy analysis performed in the center of the BaTiO_3_ grains and at their edges. The surfaces of large BT nanoparticles are decorated with very fine spherical Ag nanoparticles. Both components show a lognormal-type of size distribution (Figure 2c,d), BaTiO_3_ having diameters in the range of 10–120 nm with the maximum at 60 nm, while Ag nanoparticles have diameters in the range of 1–17 nm, with a maximum around ~3 nm. The high crystallinity degree of the hybrid nanoparticles is revealed by the large and bright spots composing the concentric diffraction rings in the Selected Area Electron Diffraction (SAED) patterns (Figure 2e). According to the JCPDS fiches for BaTiO_3_ with: tetragonal (JCPDS card 89-1428), cubic (JCPDS card 75-0216), or hexagonal (JCPDS card 82-1175) symmetries and for cubic Ag (JCPDS card no. 04-0783), the analysis of the spots (Figure 2e) may be assigned as follows: the spot denoted as (1) may correspond to the (002) planes of hexagonal BaTiO_3_, the spot (2) may be either assigned to (100) tetragonal or to (100) cubic BaTiO_3_, the spot (3) either to (110) cubic or to (104) hexagonal BaTiO_3_, the spot (4) may correspond to (111) cubic, (111) tetragonal, or to (006) hexagonal BaTiO_3_ or (111) of cubic Ag, due to their very similar planar distances.

Qualitative energy dispersive X-ray analysis EDX (Figure S2, Supplementary material) indicates a high purity of the hybrid inorganic powders, i.e., only the presence of the expected elements are revealed: Ba, Ti, O, and Ag from the samples and Cu and C from the microscope grid. To determine the Ag distribution in respect to the BTO nanoparticles, EDX mapping in the Scanning TEM (STEM) mode on the 2%Ag–98%BT hybrid powders was performed (Figure S3, Supplementary material). With this method, both spatial and spectral information was acquired simultaneously in each pixel. Overlapping the obtained elemental maps of Ag and Ba demonstrates that Ag nanoparticles are localized predominantly at the surface of the BT nanoparticles.

**Figure 3:**
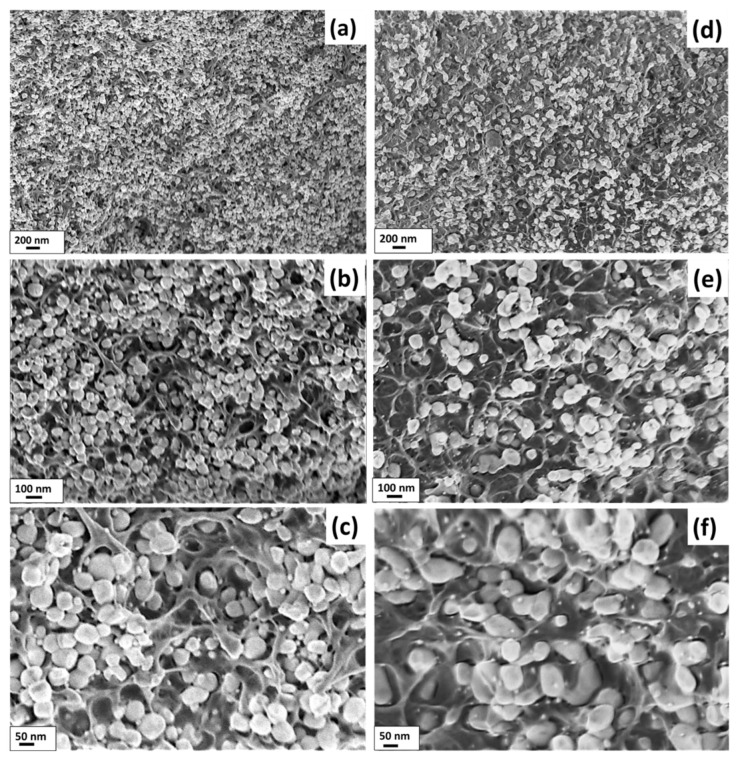
SEM micrographs with different magnifications realised in cryo-fractured fresh surfaces of 20%(*x*Ag − (1 − *x*)BT) − 80%PVDF thick films with two compositions: (**a**–**c**) *x* = 2 vol.%, (**d**–**f**) *x* = 27 vol.%.

The SEM analysis realized on the fresh cryo-fractured surfaces of 20%(*x*Ag − (1 − *x*)BT) − 80%PVDF thick film composites with extreme values of Ag fillers, *x* = 2 vol.% and *x* = 27 vol.% shown in Figure 3 at different magnifications, indicates an almost homogeneous hybrid filler distribution inside the polymer matrix. In the high-resolution micrographs (Figure 3c,f), it is clearly observed that the coarser BaTiO_3_ inorganic nanoparticles are embedded in the PVDF polymer matrix while the Ag ultrafine nanoparticles remain either attached to the BaTiO_3_ surfaces or alone in the polymer volume. There are no regions with solely agglomerated Ag nanoparticles.

### 3.2. Phase Composition Analyzed by X-ray Diffraction and Infrared Spectroscopy (FTIR and Raman)

The composite thick films with a 20 vol.% total addition of hybrid Ag-BaTiO_3_ particles in the polymer PVDF matrix was characterized by X-ray diffraction (XRD) technique, Fourier transformed infrared (FTIR) spectroscopy, and Raman analysis to find details concerning the phase composition of the starting Ag-BT particles and on the polymorphism of the PVDF phase. PVDF is a semi-crystalline polymer that may exhibit various crystalline phases characterized by different molecular arrangements, the most known being α, β, and γ polymorphs, which can be found alone or as mixtures in an amorphous molecular surrounding. Among them, the most commonly obtained phase is α-PVDF, with a TGTG’ conformation (T—trans, G—gauche molecular chain linkages), while β-PVDF with all-trans zig-zag molecular chains TTTT arrangement has the highest dipolar moment (8 × 10^−30^ Cm per repeat unit cell) and possesses stronger ferroelectricity among the other phases; the γ polymorph (TTTGTTTG’) is also electroactive with a smaller dipole moment [40]. The dielectric, ferroelectric, and piezoelectric properties are enhanced by the conformations with a larger dipole moment, i.e., in the electroactive phases β and γ. The phase composition was observed to be sensitive to the processing strategy and parameters, to co-polymerisation [41,42], or by nanofiller additions [43,44].

**Figure 4:**
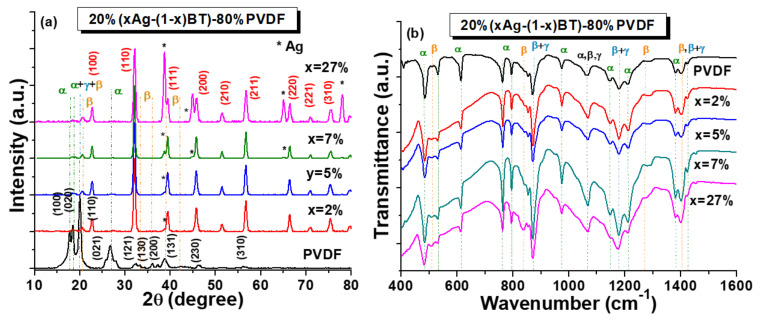
(**a**) XRD patterns and (**b**) FTIR spectra obtained for PVDF and 20%(*x*Ag − (1 − *x*)BT) − 80%PVDF composites with *x* = 2, 5, 7, and 27 vol.% Ag.

Figure 4, a presents the X-ray diffractograms of PVDF and 20%(*x*Ag − (1 − *x*)BT) − 80%PVDF flexible composites with *x* = 2, 5, 7, and 27 vol.% Ag. The XRD pattern of pure PVDF attests to the co-existence of the α, β, and γ polymorphs by the presence of the peaks at 17.7°, 18.3°, 19.9°, and 26.7° characteristic of the α phase with the diffraction planes (100), (020), (110), and (021), respectively, and the superimposed peaks from 18.5°, 19.2°, and 20.0°, which are specific to the γ phase. The β peak at 20.26° (110) is superimposed also to those of the α and γ phases, but the most relevant β peak corresponding to the (200) diffraction planes can be very well observed at 38.8° [45,46,47,48]. The X-ray diffractograms for all the PVDF composites confirmed the existence of BT and Ag phases, with a gradual increase of the main Ag peaks (denoted with *) when increasing *x*. The diffraction peaks characteristic of the Ag reflections are located at 2θ ~ 38°, 44°, 65°, and 78°, corresponding to the diffraction planes (111), (200), (270), and (311) of the cubic stable phase (JCPDS card no. 01-087-0718) [16,17]. For the BaTiO_3_ nanoparticles, there is no visible tetragonal split of the characteristic peak 2θ ~ 45° (200)/(002), indicating that the employed BT powders have a pseudo-cubic perovskite structure. The Debye Scherrer analysis of the main peaks of BT and Ag provided values for the crystallite sizes of ~8 nm for Ag and ~67 nm for BT which are in the range of the grain sizes observed by TEM analyses and confirming the predominant single-crystalline nature of both types of inorganic nanoparticles used as fillers. It is worth noting that for fine BaTiO_3_ particles, the cubic state was traditionally considered the main polymorph, due to the impossibility of detecting the tetragonal splitting around 2θ ~ 45°, typical of coarse grains or ceramics at room temperature [47]. However, recent studies using high-resolution structural analyses have shown that not only cubic, but other symmetries such as tetragonal or orthorhombic, have very small distortions or even hexagonal symmetry or often superpositions of more polymorphs as a result of surface relaxations, anisotropy, or nanoscale defects obtained in high purity ultrafine BT particles [49,50,51,52,53]. Corroborating the room temperature XRD data obtained with laboratory equipment and the results of TEM analyses, it can be inferred that an average pseudo-cubic state, as detected by the structural analysis, can comprise predominant cubic BT nanoparticles, together with ones showing other symmetries and small distortions, or the possible coexistence of tetragonal, hexagonal, or other symmetries at the nanoscale. The successful synthesis by a low thermal budget method of fine single-crystalline Ag nanoparticles onto the BT templates whose size and structure remain unchanged is thus demonstrated. To reveal the possible modifications of PVDF crystalline structure induced by the addition of inorganic Ag-BT hybrid filler in the composite material, qualitative infrared spectroscopy analysis by FTIR and Raman was further performed.

The FTIR spectra of pure PVDF and PVDF flexible composites presented in Figure 4, b confirmed the observed mixture of polymorphs as determined from XRD analysis. The characteristic peaks assigned to the nonpolar α-PVDF phase are located at 490, 532, 614, 763, 795, 855, 973, 1148, 1212, 1382, and 1423 cm^−1^ with the most representative intense peaks at 763 and 614 cm^−1^, while the peaks specific to the ferroelectric β polymorph reported at 510, 840, 1275, and 1402 cm^−1^ wavenumbers, as validated in [41,47]. However, the literature reports and our FTIR data have shown that there are also peaks in the range of 870–885 cm^−1^, 1171–1182 cm^−1^, and 1398–1404 cm^−1^ which have similar characteristics to the α, β, and γ-phases or to mixed phases [19,34,35,36,37,41,54,55,56,57]. The most important bands are specific to the α and β phases, whose formation is dependent on the processing conditions and composition. It is observed that by incorporation of 20 vol.% quantities of hybrid *x*Ag − (1 − *x*)BT fillers, a change in the PVDF crystal phase composition is induced, i.e., an increase of the polar β-PVDF phase is acquired. The highest addition of silver *x* = 27 vol.% seems to enhance the main bands (840 and 1402 cm^−1^), thus demonstrating that the formation of the β phase in the PVDF composite materials is promoted by the presence of ultrafine silver nanoparticles. 

**Figure 5:**
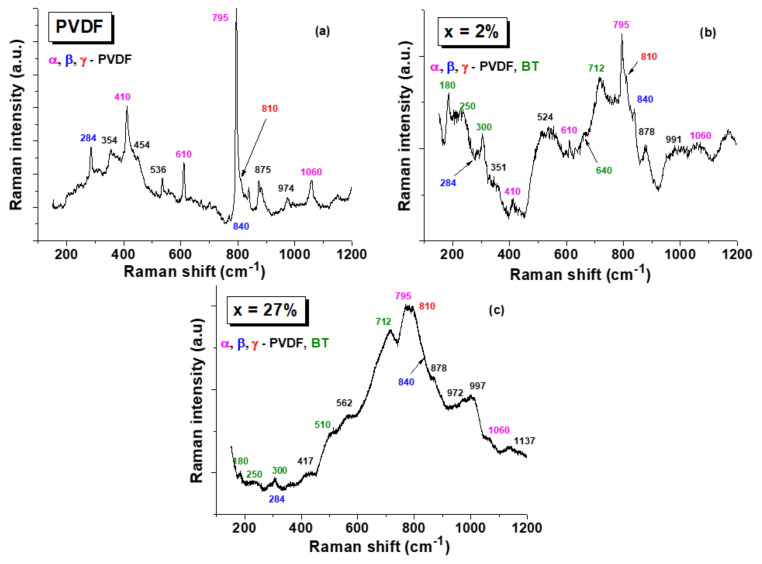
Room temperature Raman spectra of PVDF thick film (**a**) and of 20%(*x*Ag − (1 − *x*)BT) − 80%PVDF composites with *x* = 2% (**b**) and *x* = 27% (**c**) in the frequency range of 100 cm^−1^ to 1200 cm^−1^.

Another complementary infrared spectroscopy characterization to acquire information concerning the crystalline phases in the PVDF-based flexible hybrid composite is the Raman analysis. The room temperature Raman spectra of PVDF thick film and the 20%(*x*Ag − (1 − *x*)BT) − 80%PVDF composites with two extreme compositions: *x* = 2 vol.% and *x* = 27 vol.%, shown in Figure 5, allowed the identifications of the major Raman modes characteristic to the PVDF polymer matrix [58]. It can be observed that the predominant polymorph is the α-phase (identified by its major sharp peak at 795 cm^−1^ and by smaller peaks at 410 cm^−1^, 610 cm^−1^, and 1060 cm^−1^ indicated with the pink color in the figure), together with amounts of ferroelectric polar phases: β-PVDF with the main peaks at 284 cm^−1^ and 840 cm^−1^ (denoted by the blue color), and the γ-phase identified by a small peak at 810 cm^−1^ (red color in Figure 5), according to the band assignment proposed in the [13,57,59,60,61] and to the results obtained using the FTIR characterization. For the composition with the small Ag addition of *x* = 2 vol.% (Figure 5b), the Raman spectra show the specific modes of the PVDF matrix mentioned before, with a predominant α—phase and ones of the BaTiO_3_ filler: a positive peak/dip around 180 cm^−1^, possibly related to the interference between the damping of A1(TO) phonon and orthorhombic features around 190 cm^−1^ [62,63,64], A1(TO1) stiffened the component of the soft mode at 250 cm^−1^, E+B1 exhibited a sharp peak infrared inactive („silent mode”) at 300 cm^−1^, A1(TO4) at 510 cm^−1^, and the high-frequency LO4 band at 712 cm^−1^ which are considered signatures of the long-range ferroelectric tetragonal phase of BaTiO_3_ [62,63,65]. Additionally, a small peak around 640 cm^−1^ (Figure 5b) indicates either traces of hexagonal symmetry of BaTiO_3_ or internal defects in the BaTiO_3_ structure [66]. Therefore, according to the present structural and microstructural analysis, BaTiO_3_ is clearly not in a pure cubic state (in which the Raman activity is forbidden), but in an average pseudo-cubic state as found by the large-scale XRD analysis, while at the local scale, other polymorphs as orthorhombic or hexagonal in the BaTiO_3_ fine powders are likely, besides the tetragonal phase, all of them characterized by the very similar interplanar distances detected. Similar findings have also been reported in the literature for fine BaTiO_3_ powders [39,64,66]. When the concentration of Ag nanoparticles is higher, the Raman intensity strongly increases as a result of the surface-enhanced Raman scattering process [61,67]; the specific modes of both BaTiO_3_ and PVDF are strongly smeared in the whole frequency range and the α-phase is predominantly maintained, together with the other polymorphs which are also present (Figure 5c). Based on the overall data, we can conclude that by increasing the Ag-BT hybrid filler addition in the composites, the formation of the polar structure β and γ phases of PVDF is promoted, although the major phase remains the α-polymorph. An increase of the electroactive β and γ phases of PVDF as induced by the presence of silver inclusions was also reported in recent literature [43,44]. A detailed nanoscale experimental study combined with DFT calculations demonstrated that the mechanism favoring the formation of polar phases resulted from a polar interfacial coupling between the filler and the molecular chains of the polymer promoting specific polar arrangements, i.e., the presence of electroactive phases [44]. The structural modifications induced by the presence of an inorganic hybrid filler are expected to support a permittivity increase and in general, to enhance ferro-, pyro-, and piezoelectric responses in the flexible composites concerning the characteristics of the pure PVDF material.

### 3.3. Electrical Properties and Mechanical Harvesting Responses

#### 3.3.1. Dielectric, Conductive, and Ferroelectric Properties

The dielectric properties are expected to be affected by the presence of an inorganic hybrid filler, first of all, because of the addition of high permittivity dielectric BaTiO_3_ particles in the low-permittivity polymer matrix, as predicted by effective field models [68,69] and by finite element calculations [70,71]. Room temperature permittivity of nanostructured BaTiO_3_ is in the range of 800–1000 for dense nanoceramics with a grain size of about ~50 nm [72,73], while for BaTiO_3_ nanopowders with a similar diameter range, the permittivity was not reported because its direct measurement or evaluation is subjected to large errors and is dependent on the adopted model for estimation [48]. Nevertheless, the permittivity in ferroelectric powders is more than one order in magnitude higher than that of polymers and would cause an increase in the effective permittivity in composites. Moreover, embedding highly conductive (e.g., noble metal nanoparticles) filler into the polymer matrix leads to a further permittivity increase as a result of the dielectric material (polymer) confinement between the metallic particles, thus creating local capacitors whose total effect is to provide an equivalent larger capacitance and therefore, a higher equivalent material permittivity. This effect was also demonstrated numerically by using finite element calculations in previous work [30].

**Figure 6:**
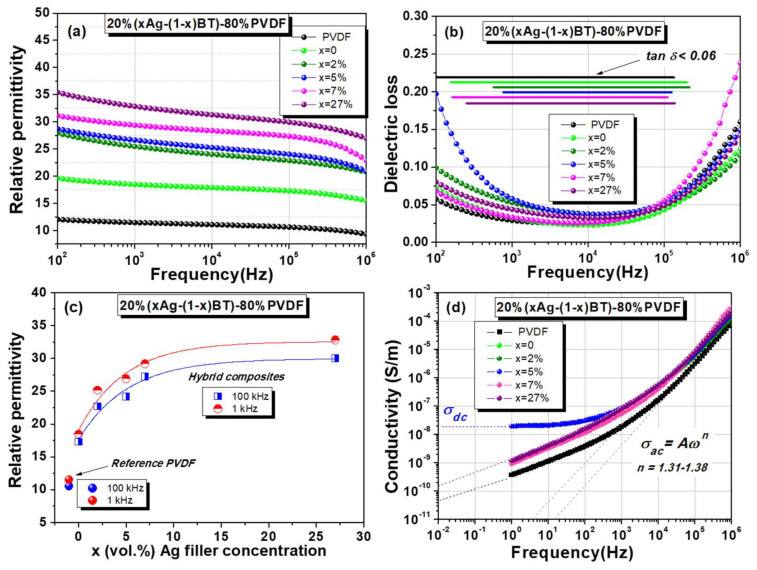
Room temperature dielectric properties of 20%(*x*Ag − (1 − *x*)BT) − 80%PVDF flexible composites as a function of frequency: (**a**) Relative permittivity; (**b**) Dielectric loss (tanδ) (the frequency range for which tanδ≤0.06 is shown, for each composition); (**c**) Relative permittivity vs. Ag filler concentration (the lines are guides for the eye) (*x*); (**d**) Conductivity vs. frequency in log-log scale.

Room temperature impedance spectroscopy analysis provided the frequency-dependence of permittivity (Figure 6a) and tangent loss (Figure 6b). The beneficial impact of the addition of inorganic filler resulting in a permittivity increase is clearly observed at any frequency. For example, at 1 kHz, the relative permittivity increases from ~11.6 (PVDF) to ~18.5 for 20%BT–80%PVDF, which shows the important role of the high-permittivity BT nanoparticle addition. Further, the role of silver nanoparticles in improving the effective permittivity for a fixed value of the filler addition (20 vol.%) is clearly demonstrated, i.e., the permittivity increases when the Ag concentration *x* is larger, reaching the maximum value of ~32.8 for *x* = 27 vol.% Ag, i.e., three times larger than in the pure polymer. The permittivity rise is faster at low levels of Ag additions (*x* < 7 vol.%) and tends to stabilize for *x* = 27 vol.%, as shown in Figure 6c. It is important to mention that the dielectric losses tanδ are similar in the composites with ones of the pure polymer (below 25% in the frequency range of 10^2^–10^6^ Hz), meaning that the hybrid filling is not detrimental from the point of view of losses and all the compositions show a similar spectral range (shown in Figure 6b) for which their losses are very small: tanδ < 0.06.

All the compositions present a similar trend in their permittivity dependences vs. frequency, i.e., a gradual reduction with increasing frequency, which is similar to behavior as ones reported in [13,74,75]. Two spectral regions are accompanied by increased dielectric losses: below 1 kHz, where also permittivity increases, and above 10 kHz, where permittivity shows a faster drop at higher frequencies. These features indicate that during the regular frequency dispersion taking place in the overall investigated frequency range, two different relaxation processes are overlapped. A detailed study of the dielectric dispersion in the present PVDF-based composites should be performed in a broad temperature range; such a study is beyond the scope of this paper. In studies concerning similar PVDF-based composites, the high-frequency relaxation around room temperature in PVDF-based composites was usually assigned to the C-F dipolar intrinsic contributions due to the molecular motions of PVDF chains in the crystalline regions of the polymer and are observed irrespective of the presence of filler while the low-frequency phenomena are a combination of slow dielectric dispersion with conductivity relaxation, being mostly determined by the movement of uncompensated charges located at the interfaces [17,76,77]. In such materials, the interfacial structures contributing to the low-frequency electrical properties may be the interfaces separating the crystalline and amorphous phases of PVDF, the interfaces separating dissimilar materials (BT-polymer, Ag-polymer, Ag-BT), and polymer-air interfaces at pores or cracks accidentally formed inside the composite during processing. The low-frequency phenomena are influenced by the nature, composition, and distribution of fillers, but also by the samples’ quality, which should be very well controlled. The conductivity values increase with about one order in magnitude in the low-frequency region in the composites with respect to the pure PVDF (Figure 6d) and their values are still decreasing below the extrapolated value of 10^−9^ S/m when reducing the frequency, similar to that reported in [17]. An exception among the present 20%(*x*Ag − (1 − *x*)BT) − 80%PVDF compositions is *x* = 5 vol.%, for which a stabilized low-frequency conductivity is observed can be assigned to a dc value $\sigma_{dc}=$2 × 10^−8^ S/m. Due to the conductivity relaxation, this composition also shows the highest low-frequency dielectric loss, the smallest frequency range with dielectric losses below 0.06, and the highest imaginary part of permittivity. Above 1 kHz, the ac conductivity vs. frequency is similar in all the composites (Figure 6d), according to the universal dynamic law: $\sigma_{ac}=A\omega^{n}$ [78] with an almost similar power coefficient *n* = 1.3–1.38 for all the compositions, which usually describes a hoping conductivity process [79].

**Figure 7:**
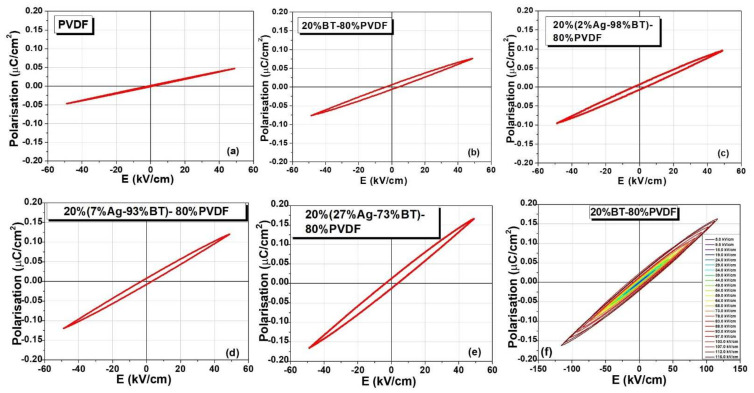
Comparative polarization-field loops at the same field amplitude of 50 kV/cm for the 20%(*x*Ag − (1 − *x*)BT) − 80%PVDF flexible composites: (**a**) PVDF, (**b**) *x* = 0, (**c**) *x* = 2 vol.%, (**d**) *x* = 7 vol.%, (**e**) *x* = 27 vol.%, and (**f**) *P*(*E*) for the maximum applied field of 116 kV/cm for the composition *x* = 0.

The polarisation-field *P*(*E*) dependences in the dynamic regime could be collected only in the sub-switching range (Rayleigh behavior). Similar field amplitudes of about 50 kV/cm loops are comparatively shown in Figure 7a–e. With exception of the lossy sample with composition *x* = 5 vol.% that did not show a proper response due to its higher dc conductivity, the other composites had breakdown fields in the range of 50–120 kV/cm and their initial permittivity reflects the same trend of increasing when increasing *x*, as found by the impedance spectroscopy study (shown in Figure 6c). No systematical decrease of the breakdown field was observed when including the hybrid filler and the composition that supported the highest applied field was 20BT-80%PVDF (Figure 7f). Even with this composition, no polarization switching was detected until its breakdown field and the recorded *P*(*E*) show only a slightly non-linear Rayleigh behavior. It is concluded that the permittivity enhancement produced by the hybrid filler addition and, in particular, by the presence of silver nanoparticles in these composites, is significant with respect to the best values reported in the literature [80] in spite of their modest storage capacity because they are not able to sustain the application of large fields. On this aspect, microstructural improvement by optimizing the processing parameters (in order to remove possible porosity, cracks, and filler agglomerations and to improve the sample homogeneity) might be further necessary in order to allow the composites to sustain the application of at least one order in magnitude higher fields. Nevertheless, their dielectric performances are similar to ones of other flexible materials proposed for mechanical-electrical energy conversion systems [6,11,15,18,19,20,21,22,23,24,25,26,27] whose permittivity increase due to the addition of BT and mostly of Ag nanoparticles is advantageous with respect to the permittivity of a few units as typically shown by pure polymers.

#### 3.3.2. Mechanical Energy Harvesting

Considering the recent interest in developing flexible pressure sensors for wearable devices, health and human motion monitoring, and energy scavenging systems, the flexible composites were further tested in order to find out if the presence of the Ag-BT hybrid filler would improve their mechanical-to-electrical conversion, i.e., their harvesting capability. It was shown in recent reports that the presence of fillers embedded into polymer matrix improves the piezo- and triboelectric responses [11,81]. The first tests were realized in a capacitive configuration under a regular mechanical loading regime by using a periodical strain uniformly applied on the superior sample surface (with impact energy of *W* = 9.07 mJ and a hit rate frequency of 1.71 Hz) and using the set-up described in Figure S1, Supplementary material.

**Figure 8:**
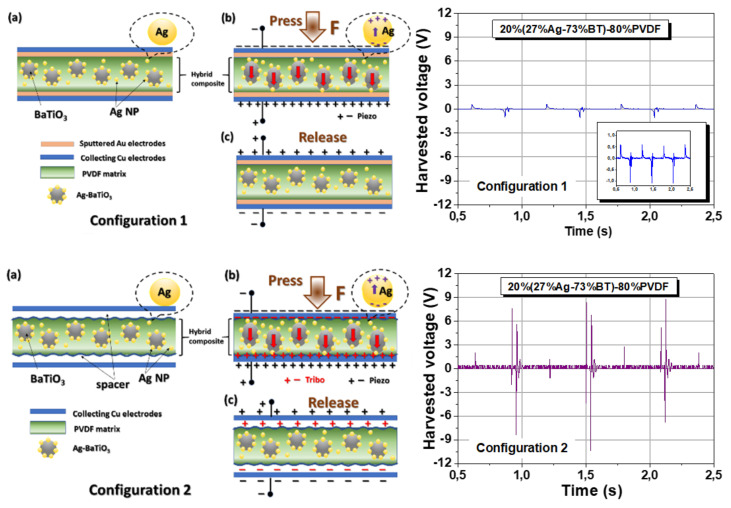
Schematic representation of the mechanical energy harvesting experiments in two configurations: Configuration 1 (**a**–**c**) when the flexible film is placed inside the collecting capacitive device in contact with the collecting Cu electrodes, and Configuration 2 (**a**–**c**) when not electrode plated composite thick film is freely placed inside the collecting electrodes. The proposed mechanism Configuration 1 and 2 of the mechanical energy harvesting (**left**) and their corresponding voltage outputs measured in open circuit conditions (**right**).

In the first set of experiments (Figure 8: Configuration 1, a), the flexible thick film composite was electroplated with gold by rf-sputtering and then placed inside the collecting capacitive device, in contact with the collecting Cu electrodes. In the second series of experiments (Figure 8: Configuration 2, a), the composite thick film was not plated at all (it had free surfaces) and was freely placed inside the collecting electrodes so that an air spacer of about 1mm was formed between the collecting electrodes and the composite-air interfaces. By pressing the superior electrode with the same periodical impact energy (Figure 8, Configurations 1–2, b) and then releasing (Figure 8, Configurations 1–2, c), it was observed that the harvested electrical output collected in open circuit conditions by the oscilloscope has very different amplitudes, i.e., a peak-to-peak value of ~1.32 V_pp_ for Configuration 1 in comparison with a much higher value of ~14.4 V_pp_ for the Configuration 2 (Figure 8). According to literature data [37], the electrical response obtained by converting the mechanical energy in a device with such configuration in which a single material is employed comprises both piezoelectric and triboelectric contributions (Configuration 2), in which the triboelectrification is enhanced by the existence of free interfaces between the composite and collecting electrode forming an air spacer [82]. Therefore, in Configuration 2, a PTENG-like composed response is generated. By collecting the charges directly from the electrodes in contact with the sputtered electrodes attached to the sample in Configuration 1, the triboelectric response is minimized by the lack of a large interface area, and therefore, the output is mainly produced by piezoelectric conversion (PENG), which has a much smaller amplitude with respect to a TENG output, as already mentioned [83,84,85]. However, it is important to infer that not only the composite film-air interface creates a triboelectric response, but also internal friction between the interfaces separating dissimilar materials inside the composite. Therefore, the output from Configuration 1 is majority piezoelectric, but not fully piezoelectric. It is worth mentioning that the presence of a piezoelectric character was also confirmed for all the non-poled composites by nanoscale piezoresponse force microscopy mapping of their surfaces, showing a clear phase and amplitude contrast for all the compositions (Figure S4, Supplementary material). 

In both configurations, the collected outputs are similar (with exception of their amplitude which differs). The collected signal has a complex aspect, is periodical, non-symmetric, and shows damping effects with different relaxation times which are dependent on the sample composition, its geometrical characteristics, device configuration, and collecting resistance. 

**Figure 9:**
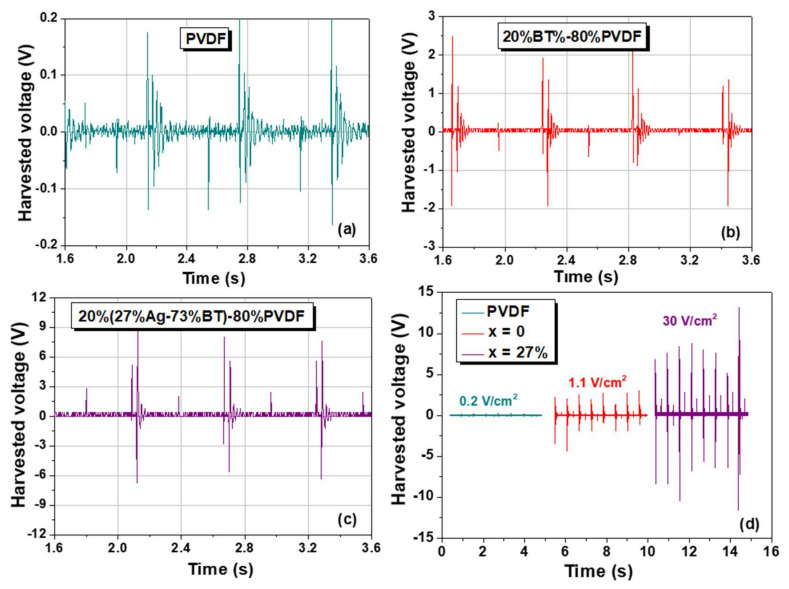
Piezoelectric energy harvesting performance for (**a**) pure PVDF, (**b**) 20%BT–80%PVDF (vol.) nanocomposite, (**c**) for 20%(27%Ag–73%BT)–80%PVDF (vol.) hybrid nanocomposite, and (**d**) a comparison between the responses of the three types of flexible thick films.

Further, Configuration 2 ensures a substantial response was used to compare the influence of BT and Ag-BT fillers into the PVDF matrix. The harvested responses of the pure PVDF, 20%BT–80%PVDF, and, of the composition with the maximum silver addition, 20%(27%Ag–73%)BT–80%PVDF composite thick films are presented and compared in Figure 9. All the flexible samples show a periodical voltage response with the same frequency as the applied mechanical testing and contain damped oscillations and periodical overdamped components. The qualitative examination of the relaxation time corresponding to the amplitude reduction indicates that the characteristic time is reduced when including 20 vol.% BT filler with respect to the case of PVDF and becomes even smaller for *x* = 27 vol.% Ag. The present results were collected using collecting load resistances of 10 MΩ, which is not yet the optimum matched load resistance to ensure a maximum harvesting output (Figure S5, Supplementary material); this aspect should be investigated in the future. A detailed study of the harvesting response with signal analysis as a function of mechanical excitation, its input energy and frequency, collecting resistance, sample geometry, its surface, and size, deserves a detailed analysis for all the compositions to reveal the role of inorganic insulating BT nanofiller and the metallic Ag nanoparticles.

The generated output voltage amplitude increases when including BaTiO_3_ filler nanoparticles with respect to the response of pure PVDF, from an amplitude of 0.2 V (PVDF) to 2–4 V (*x* = 0), but the increase is even more significant when including hybrid fillers with Ag nanoparticles: it reaches the amplitude of 9–10 V for *x* = 27 vol.% Ag hybrid nanocomposite. Concerning the maximum output voltage, these are among the highest values reported in recent literature for similar flexible composite materials [7,81,85]. Since the harvested voltage response is also dependent on the sample surface, the normalized values were determined to compare the three compositions and they are 0.2 V/cm^2^ (pure PVDF), 1.1 V/cm^2^ in 20%BT–80%PVDF nanocomposite (5.5 times higher than in PVDF), and 30 V/cm^2^ for the 20%(27%Ag–73%BT)–80%PVDF nanocomposite (150 times higher than in pure PVDF). Results indicate that for the same filler concentration of 20%, the presence of 27%Ag nanoparticles is highly beneficial and increased the output signal by a factor of ~27 times with respect to the 20% addition of only BT nanoparticles. 

As presented by various reviews mentioned in the introduction, the mechanism of combined tribo- and piezoelectric generation in the same material is not yet fully understood. Some publications suggested a few possible mechanisms to explain the beneficial role of the inorganic fillers (ferroelectric and metallic) in enhancing the output in composites with respect to ones of the pure polymer produced in the same conditions [28,29,31]. The improved output characteristics resulted in both PENG and PTENG configurations induced by the presence of inorganic fillers in the present composites with respect to the values of pure PVDF are first related to the beneficial role of BT ferroelectric nanofiller producing more electrical dipoles with a higher dipole moment (related to its ferroelectric spontaneous polarisation) inside the flexible thick film, which enhances the piezoelectric volume response and creates a larger specific interface between the PVDF matrix and ferroelectric filler (thus contributing also to augment the triboelectric output). The conductive Ag phase first provides conductive channels when they are in contact (partial percolation), resulting in more electrical charge generation [85]. Both types of fillers, because they are very fine, promoted the formation of the polar β phase of PVDF characterized by a higher dipole moment, although the α-PVDF phase still remains predominant. On the other hand, under the influence of local field inside the composite volume generated via the piezoelectric effect under pressing, the free charges migrate the length of the Ag nanoparticles when they are perfectly isolated into the dielectric matrix and create additional local dipoles (as represented schematically in Figs. 8 in both configurations). With respect to the pure polymer, all these dipoles would enhance permittivity, piezoelectric response, and triboelectric charge generation. The field concentration in some regions around the interfaces between the BT, and mostly the Ag inclusions, and polymer matrix generated as a result of the electrical boundary conditions [28,30], playing a positive role in increasing the piezoelectric response and creating a kind of local self-poling (i.e., without the need of an external poling [31]), as well as the triboelectric charge generation in such composites. Both Ag and BT fillers create rough external surfaces (as seen in the comparative AFM topographical images from Figure S4, Supplementary material), thus promoting a larger friction interface for triboelectrification under the mechanical load and this is a major effect for enhancing the mechanical harvested output in the Configuration 2 with respect to the Configuration 1 (Figure 8). Finally, the addition of both types of fillers causes an enhanced permittivity, thus creating a larger capacitance of the overall device. Since both piezo/and triboelectric charges, when produced, have to first be stored for a while by the composite material itself, and then released into the external circuit and collected during the contact-separation mode, the increased capacitance is beneficial for improving the total harvesting response [32,34].

For comparison, the output currents generated in the present conditions are in the range of 0.6–1.15 μA, and the maximum harvested surface power density determined in the described experimental conditions of Configuration 2 for the sample with the composition 20%BT–80%PVDF is in the range of (0.3, 1) μW/cm^2^ and of (9–30) μW/cm^2^ for 20%(27%Ag–73%BT)–80%PVDF. Both values are superior with respect to 0.122 μW/cm^2^ reported for the modified BT-PVDF piezoelectric sensor in [11] or of 1 μW/cm^2^ [21]. They are in the same range as reported for (Ba,Ca)(Ti, Zr)O_3_-PVDF of 16.7 μW/cm^2^ [86], but lower than for hybrid PTENG reported in [62] for a complex composite formed by hybrid TiO_2_ nanosheets with Ag nanoparticles embedded into a polydimethylsiloxane matrix (48 μW/cm^2^) and with a few orders in magnitude lower than one of the highest power density of 1.21 W/m^2^ reported for surface- and pore-engineered BT-polymer composites in [34]. We may conclude that the present harvesting outputs found in BT-PVDF, and in particular in (Ag-BT)-PVDF hybrid composites produced by a simple wet synthesis method, are very promising and their response can be further improved and optimized.

## 4. Conclusions

The modifications of the structural, dielectric, and piezoelectric properties of flexible PVDF-based composites induced by the addition of a total amount of 20 vol.% hybrid fillers composed of variable quantities of Ag and BaTiO_3_ nanopowders are presented. Thick films with formula 20%(*x*Ag − (1 − *x*)BaTiO_3_) − 80%PVDF (*x* = 0, 2, 5, 7 and 27 vol.%) composed by ultrafine (~3 nm) silver nanoparticles directly precipitated onto BaTiO_3_ (~60 nm average diameter) nanoparticles were realized by solution casting. The polar β phase of PVDF is enhanced by the presence of filler, although the α-PVDF phase remains predominant in all the compositions. A substantial permittivity increase, frequency stable, from $\varepsilon_{r}$ ~ 11 (PVDF) to $\varepsilon_{r}$ ~ 18 for *x* = 0 and further to $\varepsilon_{r}$ ~ 33 for (*x* = 27 vol.%), (measured at 1 kHz) was reached, with a good dielectric character and low losses. This is one of the highest permittivity augmentations obtained for percolated compositions. This result is due to the local field enhancement in the regions close to the filler-PVDF interfaces, which is additionally intensified by the presence of silver nanoparticles. The addition of Ag nanoparticles is also beneficial for the mechanical harvesting ability and causes a substantial increase of the voltage output: the amplitude of the maximum harvested output increases from ~0.2 V/cm^2^ (PVDF) to ~1.1 V/cm^2^ for *x* = 0 and reaches ~30 V/cm^2^ for *x* = 27 vol.% Ag as result of a mixed piezoelectric response and triboelectric charge generation. In conclusion, the strategy of decorating the BaTiO_3_ surfaces with small amounts of fine Ag nanoparticles below 27% for producing hybrid nanofillers in polymer matrices provides frequency-stable high permittivity and substantial mechanical-electrical conversion responses and should be further explored and optimized.

## Data Availability

The data presented in this study are available on request from the corresponding author.

## Supplementary Materials

The following supporting information can be downloaded at: https://www.mdpi.com/article/10.3390/nano12060934/s1, Figure S1: Block diagram of the piezoelectric harvesting tester, Figure S2. Example of an energy dispersive X-ray analysis (EDX) spectrum, collected on hybrid Ag-BaTiO_3_ nanoparticles agglomerates, Figure S3. High Angle Annular dark-field (HAADF) image and EDX-STEM elemental maps showing the spatial distributions of the Ag and Ba in 2%Ag–98%BT hybrid powders, on some aggregated larger BT grains, Figure S4. Table showing the AFM topography and local piezoelectric contrast for the flexible composites with the indicated compositions, Figure S5. Peak-to-peak harvested voltage for a sample with the composition of 20%BT–80%PVDF in the range of 2–10 MΩ.

## References

[1] Xu M., Kang H., Guan L., Li H., Zhang M. (2017). Facile Fabrication of a Flexible LiNbO_3_ Piezoelectric Sensor through Hot Pressing for Biomechanical Monitoring. CS Appl. Mater. Interfaces.

[2] Jing Q., Kar-Narayan S. (2018). Nanostructured polymer-based piezoelectric and triboelectric materials and devices for energy harvesting applications. J. Phys. D Appl. Phys..

[3] Zha J.W., Zheng M.S., Fan B.H., Dang Z.M. (2021). Polymer-based dielectrics with high permittivity for electric energy storage: A review. Nano Energy.

[4] Xin Y., Sun H., Tian H., Guo C., Li X., Wang S., Wang C. (2016). The use of polyvinylidene fluoride (PVDF) films as sensors for vibration measurement: A brief review. Ferroelectrics.

[5] Chen X., Han X., Shen Q.D. (2017). PVDF-Based Ferroelectric Polymers in Modern Flexible Electronics. Adv. Electron. Mater..

[6] Guo S., Duan X., Xie M., Aw K.C., Xue Q. (2020). Composites, Fabrication and Application of Polyvinylidene Fluoride for Flexible Electromechanical Devices: A Review. Micromachines.

[7] Li R., Zhao Z., Chen Z., Pei J. (2017). Novel BaTiO_3_/PVDF composites with enhanced electrical properties modified by calcined BaTiO_3_ ceramic powders. Mater. Express.

[8] Dang Y.M., Zheng M.S., Zha J.W. (2018). Improvements of dielectric properties and energy storage performances in BaTiO_3_/PVDF nanocomposites by employing a thermal treatment process. J. Adv. Diel..

[9] Hu X., Yan X., Gong L., Wang F., Xu Y., Feng L., Zhang D., Jiang Y. (2019). Improved Piezoelectric Sensing Performance of P(VDF–TrFE) Nanofibers by Utilizing BTO Nanoparticles and Penetrated Electrodes. ACS Appl. Mater. Interfaces.

[10] Abdolmaleki H., Agarwala S. (2020). PVDF-BaTiO_3_ Nanocomposite Inkjet Inks with Enhanced Phase Crystallinity for Printed Electronics. Polymers.

[11] Yang Y., Pan H., Xie G., Jiang Y., Chen C., Su Y., Wang Y., Tai H. (2020). Flexible piezoelectric pressure sensor based on polydopamine-modified BaTiO_3_/PVDF composite film for human motion monitoring. Sens. Actuators A.

[12] Fang F., Yang W., Yu S., Luo S., Sun R. (2014). Mechanism of high dielectric performance of polymer composites induced by BaTiO_3_-supporting Ag hybrid fillers. Appl. Phys. Lett..

[13] Ghosh B., Tamayo Calderon R.M., Espinoza-Gonzalez R., Hevia S.A. (2017). Enhanced dielectric properties of PVDF/CaCu_3_Ti_4_O_12_: Ag composite films. Mater. Chem. Phys..

[14] Wang L., Huang X., Zhu Y., Jiang P. (2018). Enhancing electrical energy storage capability of dielectric polymer nanocomposites via the room temperature Coulomb blockade effect of ultra-small platinum nanoparticles. Phys. Chem. Chem. Phys..

[15] Shuai C., Liu G., Yang Y., Qi F., Peng S., Yang W., He C., Wang G., Qian G. (2020). A strawberry-like Ag-decorated barium titanate enhances piezoelectric and antibacterial activities of polymer scaffold. Nano Energy.

[16] Luo S., Yu S., Sun R., Wong C.P. (2014). Nano Ag-Deposited BaTiO_3_ Hybrid Particles as Fillers for Polymeric Dielectric Composites: Toward High Dielectric Constant and Suppressed Loss. ACS Appl. Mater. Interfaces.

[17] Silakaew K., Thongbai P. (2019). Suppressed loss tangent and conductivity in high-permittivity Ag-BaTiO_3_/PVDF nanocomposites by blocking with BaTiO_3_ nanoparticles. Appl. Surf. Sci..

[18] Askaria H., Khajepour A., Khamesee M.B., Saadatnia Z., Wang Z.L. (2018). Piezoelectric and triboelectric nanogenerators: Trends and impacts. Nano Today.

[19] Zhu J., Zhu M., Shi Q., Wen F., Liu L., Dong B., Haroun A., Yang Y., Vachon P., Guo X. (2020). Progress in TENG technology—A journey from energy harvesting to nanoenergy and nanosystem. EcoMat.

[20] Zhang D., Wang D., Xu Z., Zhang X., Yang Y., Guo J., Zhang B., Zhao W. (2021). Diversiform sensors and sensing systems driven by triboelectric and piezoelectric nanogenerators. Coord. Chem. Rev..

[21] Wang Z.L., Song J.H. (2006). Piezoelectric nanogenerators based on zinc oxide nanowire arrays. Science.

[22] Wang Z.L. (2019). Triboelectric nanogenerator boosts smart green tires. Adv. Funct. Mater..

[23] Wang Z.L. (2020). Triboelectric nanogenerator (TENG)-sparking an energy and sensor revolution. Adv. Energy Mater..

[24] Zheng Q., Zhang H., Mi H., Cai Z., Ma Z., Gong S. (2015). Fabrication of composite PVDF-ZnO nanofiber mats by electrospinning for energy scavenging application with enhanced efficiency. J. Polym. Res..

[25] Alluri N.R., Saravanakumar B., Kim S.J. (2015). Flexible, hybrid piezoelectric film (BaTi_1−x_Zr_x_O_3_)/PVDF nanogenerator as a self-powered fluid velocity sensor. ACS Appl. Mater. Interfaces.

[26] Jella V., Ippili S., Eom J.H., Choi J., Yoon S.G. (2018). Enhanced output performance of a flexible piezoelectric energy harvester based on stable MAPbI3-PVDF composite films. Nano Energy.

[27] Whiter R.A., Narayan V., Narayan S.K.A. (2014). Scalable nanogenerator based on self-poled piezoelectric polymer nanowires with high energy conversion efficiency. Adv. Energy Mater..

[28] Paik H., Choi Y.Y., Hong S., No K. (2015). Effect of Ag nanoparticle concentration on the electrical and ferroelectric properties of Ag/P(VDF-TrFE) composite films. Sci. Rep..

[29] Dudem B., Kim D.H., Bharat L.K., Yu J.S. (2018). Highly-flexible piezoelectric nanogenerators with silver nanowires and barium titanate embedded composite films for mechanical energy harvesting. Appl. Energy.

[30] Cazacu A., Curecheriu L., Neagu A., Padurariu L., Cernescu A., Lisiecki I., Mitoseriu L. (2013). Tunable gold-chitosan nanocomposites by local field engineering. Appl. Phys. Lett..

[31] Lu H., Shi H., Chen G., Wu Y., Zhang J., Yang L., Zhang Y., Zheng H. (2021). High-Performance Flexible Piezoelectric Nanogenerator Based on Specific 3D Nano BCZT@Ag Hetero-Structure Design for the Application of Self-Powered Wireless Sensor System. Small.

[32] He X., Guo H., Yue X., Gao J., Xi Y., Hu C. (2015). Improving energy conversion efficiency for triboelectric nanogenerator with capacitor structure by maximizing surface charge density. Nanoscale.

[33] Liu Z., Muhammad M., Cheng L., Xie E., Han W. (2020). Improved output performance of triboelectric nanogenerators based on polydimethylsiloxane composites by the capacitive effect of embedded carbon nanotubes. Appl. Phys. Lett..

[34] Tantraviwat D., Ngamyingyoud M., Sripumkhai W., Pattamang P., Rujijanagul G., Inceesungvorn B. (2021). Tuning the Dielectric Constant and Surface Engineering of a BaTiO_3_/Porous PDMS Composite Film for Enhanced Triboelectric Nanogenerator Output Performance. ACS Omega.

[35] Jung W.S., Kang M.G., Moon H.G., Baek S.H., Yoon S.J., Wang Z.L., Kim S.W., Kang C.Y. (2015). High Output Piezo/Triboelectric Hybrid Generator. Sci. Rep..

[36] Suo G., Yu Y., Zhang Z., Wang S., Zhao P., Li J., Wang X. (2016). Piezoelectric and Triboelectric Dual Effects in Mechanical-Energy Harvesting Using BaTiO_3_/Polydimethylsiloxane Composite Film. ACS Appl. Mater. Interfaces.

[37] Wang N., Liu Y., Ye E., Li Z., Wang D. (2022). Control methods and applications of interfacecontact electrification of triboelectric nanogenerators: A review. Mater. Res. Lett..

[38] Kim D.W., Lee J.H., Kim J.K., Jeong U. (2020). Material aspects of triboelectric energy generation and sensors. NPG Asia Mater..

[39] Lukacs V.A., Airimioaei M., Padurariu L., Curecheriu L.P., Ciomaga C.E., Bencan A., Drazic G., Avakian M., Jones J.L., Stoian G. (2021). Phase coexistence and grain size effects on the functional properties of BaTiO_3_ ceramics. J. Eur. Ceram. Soc..

[40] Abdalla S., Obaid A., Al-Marzouki F.M. (2016). Preparation and characterization of poly(vinylidene fluoride): A high dielectric performance nano-composite for electrical storage. Results Phys..

[41] Martins P., Lopes A.C., Lanceros-Mendez S. (2014). Electroactive phases of poly(vinylidene fluoride): Determination, processing and applications. Prog. Polym. Sci..

[42] Vasic N., Steinmetz J., Gorke M., Sinapius M., Huhne C., Garnweitner G. (2021). Phase Transitions of Polarised PVDF Films in a Standard Curing Process for Composites. Polymers.

[43] Kim S.H., Park S.J., Cho C.Y., Kang H.S., Sohn E.H., Park I.J., Ha J.W., Lee S.G. (2019). Preparation and electroactive phase adjustment of Ag-doped poly(vinylidene fluoride) (PVDF) films. RSC Adv..

[44] Liu Y., Yang T., Zhang B., Williams T., Lin Y.T., Li L., Zhou Y., Lu W., Kim S.H., Chen L.Q. (2020). Structural Insight in the Interfacial Effect in Ferroelectric Polymer Nanocomposites. Adv. Mater..

[45] Davis G., McKinney J., Broadhurst M., Roth S. (1978). Electric-field-induced phase changes in poly(vinylidene fluoride). J. Appl. Phys..

[46] Gregorio R. (2006). Determination of the α, β, and γ crystalline phases of poly(vinylidene fluoride) films prepared at different conditions. J. Appl. Polym. Sci..

[47] Cai X., Lei T., Sun D., Lin L. (2017). A critical analysis of the alpha, beta and gamma phases in poly(vinylidene fluoride) using FTIR. RSC Adv..

[48] Brunengo E., Conzatti L., Schizzi I., Buscaglia M.T., Canu G., Curecheriu L., Costa C., Castellano M., Mitoseriu L., Stagnaro P. (2021). Improved dielectric properties of poly(vinylidene fluoride)-BaTiO_3_ composites by solvent-free processing. J. Appl. Polym. Sci..

[49] Buscaglia V., Randall C.A. (2020). Size and scaling effects in barium titanate. An overview. J. Eur. Ceram. Soc..

[50] Takeuchi T., Ado K., Asai T., Kageyama H., Saito Y., Masquelier C., Nakamura O. (1994). Thickness of Cubic Surface Phase on Barium Titanate single crystalline grains. J. Am. Ceram. Soc..

[51] Smith M.B., Page K., Siegrist T., Redmond P.L., Walter E.C., Seshadri R., Brus L.E., Steigerwald M.L. (2008). Crystal Structure and the Paraelectric-to-Ferroelectric Phase Transition of Nanoscale BaTiO_3_. J. Am. Chem. Soc..

[52] Shi C., Billinge S.J.L., Puma E., Bang S.H., Bean N.J.H., de Sugny J.C., Gambee R.G., Haskell R.C., Hightower A., Monson T.C. (2018). Barium titanate nanoparticles: Short-range lattice distortions with long-range cubic order. Phys. Rev. B.

[53] Pasuk I., Neatu F., Neatu S., Florea M., Istrate C.M., Pintilie I., Pintilie L. (2021). Structural Details of BaTiO_3_ Nano-Powders Deduced from the Anisotropic XRD Peak Broadening. Nanomaterials.

[54] Dhevi D.M., Prabu A.A., Kim K.J. (2016). FTIR studies on polymorphic control of PVDF ultrathin films by heat-controlled spin coater. J. Mater. Sci..

[55] Sui Y., Chen W.T., Ma J.J., Hu R.H., Liu D.S. (2016). Enhanced dielectric and ferroelectric properties in PVDF composite flexible films through doping with diisopropylammonium bromide. RSC Adv..

[56] Xia W., Zhang Z. (2018). PVDF-based dielectric polymers and their applications in electronic materials. IET Nanodielectr..

[57] Silakaew K., Thongbai P. (2021). Effects of sub-micro sized BaTiO_3_ blocking particles and Ag-deposited nano-sized BaTiO_3_ hybrid particles on dielectric properties of Poly(vinylidene-fluoride) polymer. Polymers.

[58] Singh P., Borkar H., Singh B.P., Singh V.N., Kumar A. (2014). Ferroelectric polymer-ceramic composite thick films for energy storage applications. AIP Adv..

[59] Mattsson B., Ericsson H., Torell L.M., Sundholm F. (1999). Micro-Raman investigations of PVDF-based proton-conducting membranes. J. Polym. Sci. A Polym. Chem..

[60] Constantino C.J.L., Job A.E., Simoes R.D., Giacometti J.A., Zucolotto V., Oliveira O.N., Chinaglia D.L. (2005). Phase transition in Poly(Vinylidene Fluoride) investigated with micro-Raman spectroscopy. Appl. Spectrosc..

[61] Stamplecoskie K.G., Scaiano J.C., Tiwari V.S., Anis H. (2011). Optimal Size of Silver Nanoparticles for Surface-Enhanced Raman Spectroscopy. J. Phys. Chem. C.

[62] Scalabrin A., Chaves A.S., Shim D.S., Porto S.P.S. (1977). Temperature dependence of the A1 and E optical phonons in BaTiO_3_. Phys. Status Solidi B.

[63] Buscaglia V., Buscaglia M.T., Viviani M., Ostapchuk T., Gregora I., Petzelt J., Mitoseriu L., Nanni P., Testino A., Calderone R. (2005). Raman and AFM piezoresponse study of dense BaTiO_3_ nanocrystalline ceramics. J. Eur. Ceram. Soc..

[64] Shiratori Y., Pithan C., Dornseiffer J., Waser R. (2007). Raman scattering studies on nanocrystalline BaTiO_3_. Part I—Isolated particles and aggregates. J. Raman Spectrosc..

[65] Ostapchuk T., Petzelt J., Savinov M., Buscaglia V., Mitoseriu L. (2006). Grain-size effect in BaTiO_3_ ceramics: Study by far infrared spectroscopy. Phase Transit..

[66] Tsuzuku K., Couzi M. (2012). In Situ Investigation of Chemical Reactions between BaCO_3_ and Anatase or Rutile TiO_2_. J. Mater. Sci..

[67] Albrecht M.G., Creighton J.A. (1977). Anomalously intense Raman spectra of pyridine at a silver electrode. J. Am. Chem. Soc..

[68] Sareni B., Krahenbuhl L., Beroual A., Brosseau C. (1997). Effective dielectric constant of random composite materials. J. Appl. Phys..

[69] Petzelt J., Nuzhnyy D., Bovtun V., Savinov M., Kempa M., Rychetsky I. (2013). Broadband dielectric and conductivity spectroscopy of inhomogeneous and composite conductors. Phys. Status Solidi A.

[70] Pascariu V., Padurariu L., Avadanei O., Mitoseriu L. (2013). Dielectric properties of PZT–epoxy composite thick films. J. Alloys Compd..

[71] Padurariu L., Curecheriu L.P., Mitoseriu L. (2016). Nonlinear dielectric properties of paraelectric-dielectric composites described by a 3D Finite Element Method based on Landau-Devonshire theory. Acta Mater..

[72] Buscaglia M.T., Buscaglia V., Viviani M., Petzelt J., Savinov M., Mitoseriu L., Testino A., Nanni P., Harnagea C., Zhao Z. (2004). Ferroelectric properties of dense nanocrystalline BaTiO_3_ ceramics. Nanotechnology.

[73] Curecheriu L., Buscaglia M.T., Buscaglia V., Zhao Z., Mitoseriu L. (2010). Grain size effect on the nonlinear dielectric properties of barium titanate ceramics. Appl. Phys. Lett..

[74] Xie L., Huang X., Li B.W., Zhi C., Tanaka T., Jiang P. (2013). Core–satellite Ag@BaTiO_3_ nanoassemblies for fabrication of polymer nanocomposites with high discharged energy density, high breakdown strength and low dielectric loss. Phys. Chem. Chem. Phys..

[75] Zhang Y.S., Wang M., Yang C., Shao Y.W., Qi X.D., Yang J.H., Wang Y. (2021). Heterogeneous BaTiO_3_@Ag core-shell fibers as fillers for polymer dielectric composites with simultaneously improved dielectric constant and breakdown strength. Compos. Commun..

[76] Zhang Y., Wang W., Zhang J., Ni Y. (2020). Dielectric relaxation processes in PVDF composite. Polym. Test..

[77] Shin H.K. (2020). Dielectric Relaxation in Polyvinylidene Fluoride (PVDF)/CsHSO_4_ Composites. J. Korean Phys. Soc..

[78] Jonscher A.K. (1992). Chapter 5 Low-frequency dispersion. Universal Relaxation Law.

[79] Psarras G.C. (2006). Hopping conductivity in polymer matrix–metal particles composites. Compos. Part A Appl. Sci. Manuf..

[80] Luo S., Yu J., Yu S., Sun R., Cao L., Liao W.H., Wong C.P. (2019). Significantly enhanced electrostatic energy storage performance of flexible polymer composites by introducing highly insulating-ferroelectric microhybrids as fillers. Adv. Energy Mater..

[81] Dashtizad S., Alizadeh P., Yourdkhani A. (2021). Improving piezoelectric properties of PVDF fibers by compositing with BaTiO_3_-Ag particles prepared by sol-gel method and photochemical reaction. J. Alloys Compd..

[82] Bai Y., Jantunen H., Juuti J. (2018). Energy Harvesting Research: The Road from Single Source to Multi Source. Adv. Mater..

[83] Yoon H.J., Ryu H., Kim S.W. (2018). Sustainable Powering Triboelectric Nanogenerators: Approaches and the Path towards Efficient Use. Nano Energy.

[84] Lapcinskis L., Malnieks K., Linarts A., Blums J., Smits K., Jarvekulg M., Knite M., Andris Sutka A. (2019). Hybrid Tribo-Piezo-Electric Nanogenerator with Unprecedented Performance Based on Ferroelectric Composite Contacting Layers. ACS Appl. Energy Mater..

[85] Sriphan S., Charoonsuk T., Maluangnont T., Vittayakorn N. (2019). High-performance hybridized composited-based piezoelectric and triboelectric nanogenerators based on BaTiO_3_/PDMS Composite film modified with Ti_0.8_O_2_ nanosheets and silver nanopowders cofillers. ACS Appl. Energy Mater..

[86] Wu Y., Qu J., Daoud W.A., Wang L., Qia T. (2019). Flexible composite-nanofiber based piezotriboelectric nanogenerators for wearable electronics. J. Mater. Chem. A.

