# Supplementary material for: Increasing Permittivity and Mechanical Harvesting Response of PVDF-Based Flexible Composites by Using Ag Nanoparticles onto BaTiO3 Nanofillers
Source: Nanomaterials (Basel). 2022 Mar 12;12(6):934. doi: 10.3390/nano12060934 (PMC8949362; doi:10.3390/nano12060934)
Supplement: Supplementary file 1 [file nanomaterials-12-00934-s001.zip › nanomaterials-1598851-supplementary.pdf]

nanomaterials

MDPI

Article

Increasing Permittivity and Mechanical Harvesting Response of PVDF-Based Flexible Composites by Using Ag Nanoparticles onto $BaTiO_3$ Nanofillers

Nadejda Horchidan [1], Cristina Elena Ciomaga [2,*], Lavinia Petronela Curecheriu [1], George Stoian [3], Mihaela Botea [4], Mihaela Florea [4], Valentin Adrian Maraloiu [4], Lucian Pintilie [4], Florin Mihai Tufescu [5], Vasile Tiron [6], Aurelian Rotaru [7] and Liliana Mitoseriu [1,*]

1 Dielectrics, Ferroelectrics & Multiferroics Group, Faculty of Physics, Al. I. Cuza University of Iasi, Bv. Carol I, no. 11, 700506 Iasi, Romania; nadejda.horchidan@uaic.ro (N.H.); lavinia.curecheriu@uaic.ro (L.P.C.)
2 Department of Exact & Natural Sciences, Institute of Interdisciplinary Research, Al. I. Cuza University of Iasi, Bv. Carol I, no. 11, 700506 Iasi, Romania
3 National Institute of Research and Development for Technical Physics, 700050 Iasi, Romania; gstoian@phys-iasi.ro
4 National Institute of Materials Physics, Atomistilor 405A, 077125 Magurele, Romania; mihaela.botea@infim.ro (M.B.); mihaela.florea@infim.ro (M.F.); maraloiu@infim.ro (V.A.M.); pintilie@infim.ro (L.P.)
5 GRADIENT Srl., Str. Codrescu, no. 17, 700495 Iasi, Romania; florintufescu@gradient.ro
6 Research Center on Advanced Materials and Technologies, Department of Exact & Natural Sciences, Institute of Interdisciplinary Research, Al. I. Cuza University of Iasi, Bv. Carol I, no.11, 700506 Iasi, Romania; vasile.tiron@uaic.ro
7 Faculty of Electrical Engineering and Computer Science & MANSiD Research Center, Stefan Cel Mare University, 720229 Suceava, Romania; aurelian.rotaru@usm.ro
* Correspondence: cristina.ciomaga@uaic.ro (C.E.C.); lmtsr@uaic.ro (L.M.)

Citation: Horchidan, N.; Ciomaga, C.E.; Curecheriu, L.P.; Stoian, G.; Botea, M.; Florea, M.; Maraloiu, V.A.; Pintilie, L.; Tufescu, F.M.; Tiron, V.; et al. Increasing Permittivity and Mechanical Harvesting Response of PVDF-Based Flexible Composites by Using Ag Nanoparticles onto $BaTiO_3$ Nanofillers. *Nanomaterials* **2022**, *12*, 934. https://doi.org/10.3390/nano12060934

Academic Editor: Emilia Iglesias Martínez

Received: 30 January 2022
Accepted: 10 March 2022
Published: 12 March 2022

Publisher's Note: MDPI stays neutral with regard to jurisdictional claims in published maps and institutional affiliations.

Copyright: © 2022 by the authors. Licensee MDPI, Basel, Switzerland. This article is an open access article distributed under the terms and conditions of the Creative Commons Attribution (CC BY) license (https://creativecommons.org/licenses/by/4.0/).

Supplementary figures

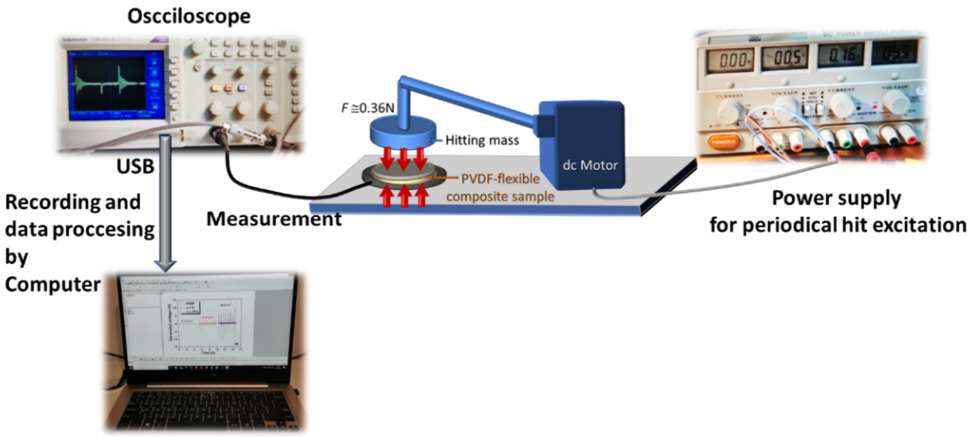

Figure S1. Block diagram of the piezoelectric harvesting tester

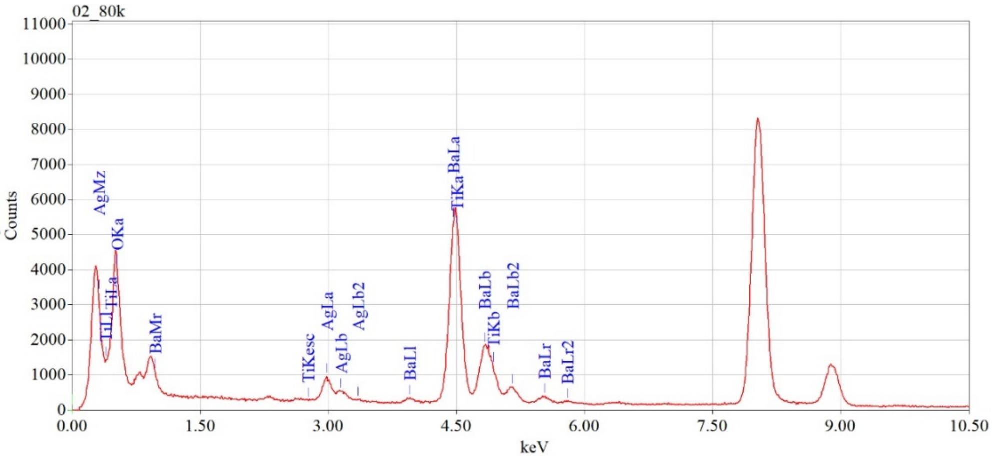

Figure S2. Example of an energy dispersive X-ray analysis (EDX) spectrum, collected on hybrid Ag-$BaTiO_3$ nanoparticles agglomerates.

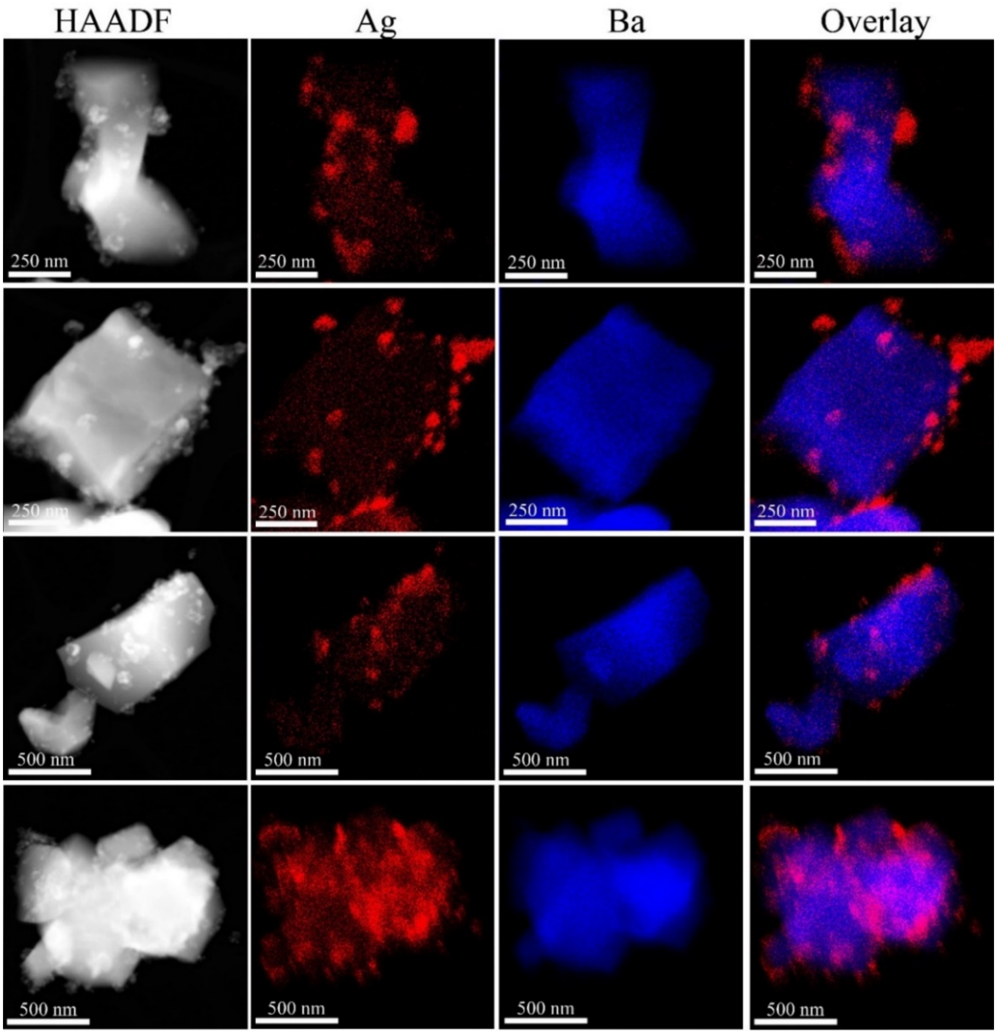

Figure S3. High Angle Annular dark field (HAADF) image and EDX-STEM elemental maps showing the spatial distributions of the Ag and Ba in 2%Ag-98%BT hybrid powders, on some aggregated larger BT grains.

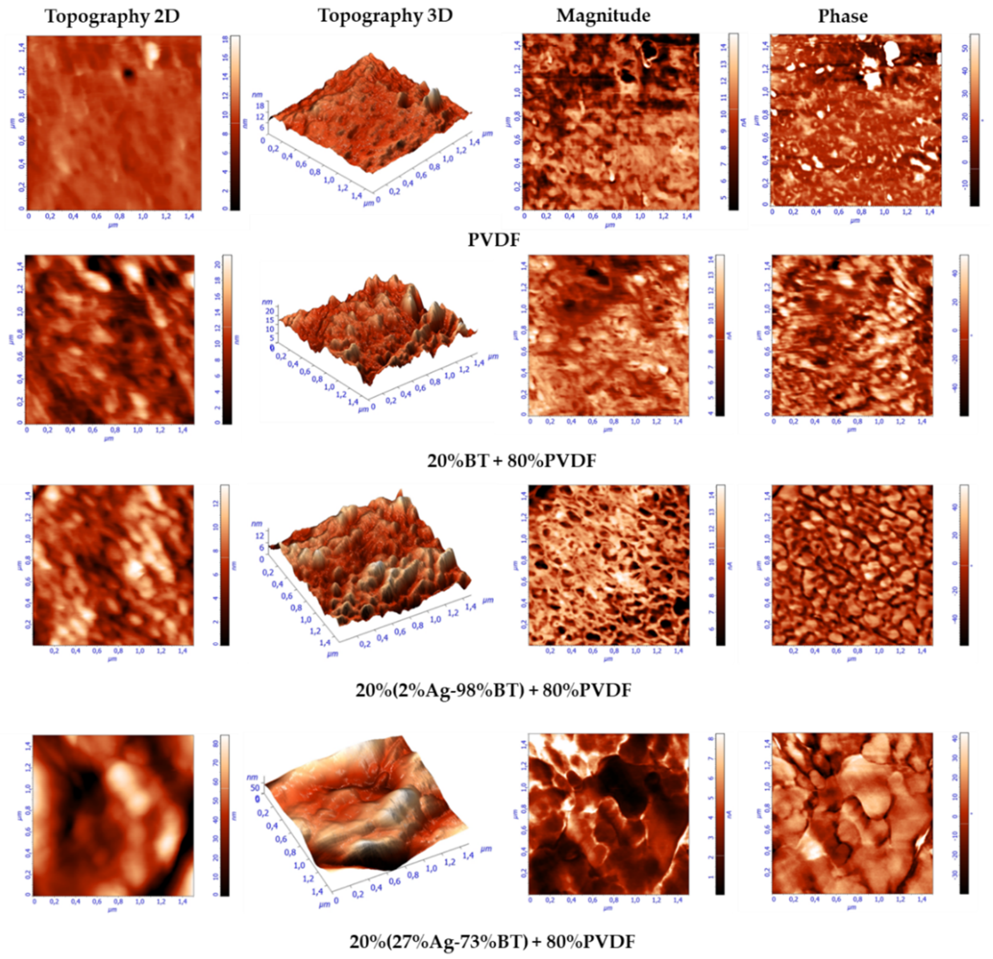

Figure S4. Table showing the AFM topography and local piezoelectric contrast for the flexible composites with the indicated compositions.

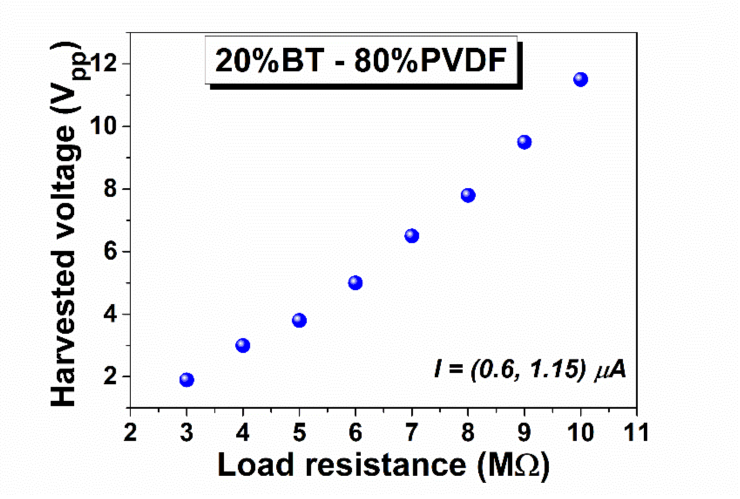

Figure S5. Peak-to-peak harvested voltage for a sample with the composition of 20%BT-80%PVDF in the range of 2-10MΩ.
